# Scarlet Fever

**Published:** 1905-02

**Authors:** Robert D. Haire

**Affiliations:** Clinton, Mo.


### SCARLET FEVER.

BY ROBERT D. HAIRE M.D.,
CLINTON, MO.

Scarlet fever is perhaps more often attended with diagnostic difficulty than any of the acute infectious diseases, owing to its many and varied types, which range from the mildest form, "scarlatina simplex" to the most malignant "scarlatina maligna." Between these extremes, an almost endless variation from the normal, shades from one to another so gradually that any sharp differentiation into groups is impossible. To this fact is also due the difficulty in distinguishing it from other contagious and eruptive fevers, as well as the non-infectious rashes caused by such drugs as quinine, belladonna, chloral and the coal tar derivatives.

From personal experience I have found the following points of diagnostic value:

Rashes, caused by drugs, are misleading, from the fact that they are ushered in by fever and appear upon the face and other locations usually characteristic of scarlet fever, so, also, are the toxic erythemata, which occur in the course of septic infection, but such cases do not present the enlargement of glands typical of scarlet fever.

In rubeola, rubella and the various forms of erythemata, if the cases are typical the diagnosis will be easily made.

The characteristic symptoms of scarlet fever are the abrupt onset, high temperature, sore throat and vomiting, with the early appearance of a rash made up of minute, brightly injected puncta, slightly elevated and closely studded together, forming a uniform or finely mottled surface, which frequently becomes confluent.

In measles the rash appears first upon the face, then spreads slowly over the body, but it is always "measley" and never confluent.

In the irregular cases, however, the diagnosis must rest upon the other symptoms, particularly those constitutional, as too much reliance upon the rash alone is always hazardous. Even desquamation is of only relative value, since dermatologists recognize the fact that it will follow any scarlatiniform erythemata which has been characterized by sufficient inflammatory change in the skin.

In this connection, however, I would impress upon you two points characteristic only of scarlet fever, viz.: The tendency of the epidermis to split just beneath the free border of the nail, then peeling off down the finger, and the persistence of the desquamation, which continues often from five to six weeks.

It is the mild form of scarlet fever which most puzzles the physician, those cases in which the fever is insignificant, the throat symptoms such as to demand little or no attention, and even the rash so slight that it may be easily classed among the minor ailments. It is to the various gradations of this class that a recent epidemic of eighteen months' duration visited our city.

During that period there were in all 110 cases reported to the health officer. Of this number there were five deaths, two due to diphtheritic complications, two to nephritis, and one, cause unknown. The epidemic displayed many phases of the disease—some were confined to their beds but a short time, or not at all, and doubtless many were never attended by a physician. The majority of cases were of moderate severity, the average period of incubation being from two to six days, while a number of cases were noted where one member of a family, constantly exposed, did not contract the disease for two weeks.

The invasion was abrupt, the average temperature ranging from 100° to 103° F., accompanied by vomiting and sore throat; in about twenty-four hours the rash appeared first upon the neck and chest, then spreading over the entire body. The highest temperature, rarely exceeding 104° F., was coincident with the full eruption, decreasing correspondingly as the rash faded. In many cases there was slight swelling of the tonsils, fauces and uvula, accompanied by pain upon swallowing.

While the fever was at its highest the patients were restless, thirsty and sometimes slightly delirious; nearly all showed marked prostration and anemia. Otitis in a mild form occurred in a number of cases, indicated by earache and discharge, but the most pronounced sequelæ of the epidemic was scarlatinal rheumatism, affecting principally the joints of the hands and feet for a few days, and a mild form of post-scarlatinal nephritis.

Since there is no specific for scarlet fever, my treatment consists in averting complications and trying to render them less severe when they do occur. Mild cases require little or no medication, as a rule they receive too much. The patient should be kept in bed and given a light diet. Milk is always to be recommended, either plain or peptonized, followed by broth, eggs and other easily digested food as improvement justifies, care being constantly exercised not to overtax the stomach, either by the quantity or quality of food.

The bowels should be first moved mildly by small doses of calomel and later kept open by enemata rather than by cathartics. Water should be given frequently and freely because of its beneficial effects upon both kidneys and bowels. In fact, it should be recognized as one of the chief therapeutic agents in the treatment of the disease.

Since a high temperature is normal in scarlet fever, it requires no special treatment unless it runs continuously above 104° F. In that event it is best reduced by means of the cold bath, cold pack or sponging, rather than by antipyretic drugs. For the country practitioner, either the cold pack or sponging will be found most expedient owing to the limitations of his environment, besides minimizing the danger from shock.

In mild or moderately severe cases stimulants are not generally required, but with the first indication of the pulse becoming weak, rapid or irregular, accompanied by a feeble heart-sound, stimulants should be given. Alcohol or strychnia in combination with digitalis may be used with advantage. The throat and nose are usually more injured than benefited by overzealous attempts at local treatment, the best results being obtained, not by active and poisonous antiseptics, but by mild, cleansing washes applied frequently and freely.

Too much stress can not be placed upon the frequent microscopical and chemical analysis of the urine during the illness and for some time afterward; even in the mild cases late nephritis is a common sequelæ. During convalescence, when necessary a tonic of iron may be used effectively. With the first signs of desquamation the skin should be bathed with warm water anointed with plain or carbolized vaseline, this treatment continuing throughout the period.

Rigid quarantine regulations should be enforced; laxity in this regard is largely responsible for the spread of the disease. In our own city the patients were isolated about thirty days when, after a house fumigation of sulphur and formaldehyde they were allowed to attend school, while many having the disease in its mildest form doubtless escaped quarantine altogether. As the disease is most contagious at the height of the febrile stage and during desquamation, complete isolation of the patient should not be less than forty days, and as much longer as there is purulent discharge from ear or nose, and until desquamation ceases.

Superfluous furniture, drapery, etc., should be removed from the sick room at the outset and the room kept as clean as possible and thoroughly ventilated. All excretia should be immediately disinfected with carbolic acid or formaldehyde, the bedclothes, linen and vessels used similarly treated before being boiled. It is a fallacy to suppose that vessels containing antiseptic fluids placed about the room will be effective in preventing the spread of the disease, and too much reliance can not be placed in fumigation by means of steam impregnated with medical agents.

The only safe prophylactic consists in burning all articles which can not be properly disinfected and boiled, then washing the walls of the room, floor and furniture with boiling water containing a solution of suphate of zinc or corrosive sublimate, 1 to 1000. Both the isolated nurse and the physician in attendance should exercise every known precaution to prevent contagion. A gown, which may be easily removed upon leaving the sick room may be used. Both should disinfect their hands and face and use daily an antiseptic gargle and nasal spray.

Bearing in mind the serious sequelæ which often attends scarlet fever, even in cases of moderate severity, and the fact that from the mildest form the most malignant may be contracted, the burden resting upon the physician in charge can not be lightly estimated. Upon him depends not only the responsibility of the patient and its future health, but the safety of the community as well. If he be conscientious in the discharge of his duty, he will see that all necessary prophylactic measures are complied with, that a rigid quarantine of not less than forty days is enforced, and that a careful examination of the patient be made before it is allowed to mingle with other children. Then, and then only, can an epidemic be averted.—*Journal Missouri State Medical Association.*

